# Effects of Foliar-Applied Mixed Mineral Fertilizers and Organic Biostimulants on the Growth and Hybrid Seed Production of a Male-Sterile Inbred Maize Line

**DOI:** 10.3390/plants12152837

**Published:** 2023-07-31

**Authors:** Riccardo Boscaro, Anna Panozzo, Simone Piotto, Selina Sterup Moore, Giuseppe Barion, Yu Wang, Teofilo Vamerali

**Affiliations:** 1 Department of Agronomy, Food, Natural Resources, Animals and the Environment, University of Padua, 35020 Legnaro, Italy; riccardo.boscaro@unipd.it (R.B.); simone.piotto.1@phd.unipd.it (S.P.); selina.moore@unipd.it (S.S.M.); giuseppe.barion@unipd.it (G.B.); teofilo.vamerali@unipd.it (T.V.); 2 Institute of Environmental Research at Greater Bay Area, Key Laboratory for Water Quality and Conservation of the Pearl River Delta, Ministry of Education, Guangzhou University, Guangzhou 510006, China; wangyu@gzhu.edu.cn

**Keywords:** *Aschophyllum nodosum* extracts, humified peat, hydrolyzed epithelium, kernel caliber, kernel production, leaf photosynthesis, root growth, *Zea mays* L.

## Abstract

Plants of inbred maize lines are characterized by low vigor due to their high rates of homozygosity and may, therefore, benefit from additional nutrients and biostimulants supplied via foliar spraying. The present study innovatively investigated the effects of foliar treatment with three commercial organic-mineral fertilizers/biostimulants on a male-sterile inbred line of maize at the five-leaf stage. The three fertilizers were characterized by their following content: (*i*) NPK + hydrolyzed animal epithelium + micronutrients (named ‘NPK + Hae + micro’), (*ii*) NK + humified peat (named ‘NK + Hp’), and (*iii*) PK + *Ascophyllum nodosum* extracts (named ‘PK + An’). The resulting shoot and root growth and seed yield and quality were compared to a control (C). Both NPK + Hae + micro and PK + An treatments enhanced root growth in the top 20 cm soil layer at the ten-leaf stage: root dry biomass increased by 80 and 24%, respectively, and the volumetric root length density by 61 and 17%. The two treatments also allowed for a larger number of commercial seeds to be produced (on average +16 bags per gross hectare vs. C) owing to a better seed caliber, which consequently reduced rates of seed disposal (−11 and −20% for PK + An and NPK + Hae + micro, respectively) and, in the case of NPK + Hae + micro, due to an increment in the number of kernels per ear (+5% vs. C). These effects were not associated with any significant changes in shoot growth, height, or leaf net CO_2_ assimilation. In this preliminary trial, peak commercial benefit was obtained with the use of hydrolyzed epithelium together with macro- and micronutrients. Further investigation into application timing and dose, and the means by which these products alleviate the effects of low vigor and stress conditions observed particularly under mechanical emasculation is, however, necessary for their full exploitation in the production of hybrid maize seeds.

## 1. Introduction

The foliar supply of nutrients is recognized as one of the most efficient agronomic practices to alleviate nutritional deficiencies in plants caused by insufficient soil availability [1]. The benefits of this practice stem from the improved nutrient uptake as compared to conventional soil-applied fertilization and the possibility of applying such a practice during critical stages of plant growth [2], regardless of soil moisture and drought conditions [3,4].

Foliar application of nutrients allows small quantities of target elements, tailored to the specific requirements of the particular crop species at different growth stages, to be supplied directly to the plant. Adequate concentrations of nutrients can improve plant nutritional status, stimulate root growth, and enhance mineral absorption from the soil solution [5]. Macro-nutrients applied through foliar spraying can directly penetrate the leaf through the external leaf cuticle or enter through the stomatal openings. However, some micronutrients, such as iron (Fe) or molybdenum (Mo), are less mobile across plant tissues. The main morpho-physiological factors affecting the dynamics of foliar nutrient absorption are leaf size, shape, and age; the presence of trichomes and waxes; and the chemical composition of the cuticle [6,7,8].

The application of plant biostimulants (PBs) represents another promising tool to enhance plant nutrition. When applied to plants, seeds, or soil, they have the ability to enhance flowering, plant growth, fruit set, crop productivity, and nutrient use efficiency (NUE). What is more, PBs have also been shown to improve tolerance against a wide range of abiotic stressors [9]. PBs consist of extremely heterogeneous substances, among which natural constituents (humic acids, protein hydrolysates, and seaweed extracts) and microbial inoculants (plant growth promoting rhizobacteria (PGPR), arbuscular mycorrhizal fungi (AMF), and *Trichoderma*) are the most commonly used. These are also associated with the greatest beneficial effects [9,10]. When in liquid suspension, microbial and non-microbial biostimulants can be applied either at soil level or via foliar spraying, and their action has been associated with several biochemical and physiological mechanisms responsible for improved plant growth and stress tolerance [11].

Initially implemented in horticulture due to the high commercial value of vegetables and fruits, foliar application of nutrients and biostimulants has been implemented at increasing rates in cereal crops such as wheat and rice in more recent years. For instance, late-season foliar N fertilization is steadily being adopted as a recommended farmer practice worldwide due to the resulting improvements in grain protein content and quality in common wheat flour and durum wheat semolina [3,12]. Among biostimulants, PGPR or plant growth-promoting fungi, such as AMF and *Trichoderma* spp., are now increasingly administered worldwide under both conventional and organic farming. Such fungi enhance the availability of different nutrients, including N, P, and micronutrients, and have also occasionally been observed to promote plant productivity [11].

To date, foliar supply of nutrients and biostimulants has been little explored in maize, often focusing on low input management systems in developing countries [13,14,15,16]. However, foliar application of nutrients and biostimulants during critical phenological stages and plant conditions has great application potential in intensive agricultural systems as well. In such systems, their application can counteract nutritional deficiencies and improve growth and development [17,18]. The chemical composition and morphology of maize leaves facilitate the absorption of supplied compounds. Indeed, the leaf shape and elevation angle allow for the fertilizers to accumulate and flow towards the leaf mid-rib and stem [19].

A further specialized application of foliar treatment with nutrients and biostimulants concerns inbred maize lines used for hybrid seed production. Here, the practice could counteract the low plant vigor associated with elevated homozygosity and improve seed production. In a previous study on inbred maize lines, foliar fertilization with N-P-K + Mg together with microelements and amino acids prevented phytotoxicity caused by post-emergence herbicides applied at the 5–6 leaf stage (V5–V6) [20]. According to the authors, foliar fertilization facilitated the metabolism of the active ingredients (a.i.) of herbicides, as revealed by improved plant biomass and elevated concentrations of antioxidants. In this regard, foliar application of target nutrients could find a relevant application in maize hybrid seed production by combining nutrients and biostimulant compounds for improved plant growth and stress tolerance. To date, the effects of different fertilizers and application doses supplied via foliar spraying on low-vigor inbred maize lines have been poorly documented. To the best of the authors’ knowledge, experimental studies documenting the use of foliar-applied fertilizers/biostimulants to counteract the low vigor of inbred maize lines are highly lacking.

Given such a background, the present study aimed to innovatively investigate the morpho-physiological effects of three commercial formulations of mixed mineral fertilizers and organic biostimulants in a non-emasculated male-sterile inbred maize line characterized by elevated homozygosity. The three products were characterized by their following content: (*i*) NPK + hydrolyzed animal epithelium + micronutrients (named ‘NPK + Hae + micro’), (*ii*) NK + humified peat (named ‘NK + Hp’), and (*iii*) PK + *Ascophyllum nodosum* extracts (named ‘PK + An’). Foliar spraying was conducted at the five-leaf stage (V5). The main objectives of the research were to verify (*i*) the physiological response by measuring the leaf chlorophyll content across the season and net CO_2_ assimilation rate one month after treatment; (*ii*) the morphological response in terms of root growth and aboveground plant biomass; and (*iii*) whether possible plant improvements translate into higher hybrid seed yields and quality in terms of kernel caliber and germinability.

## 2. Results

### 2.1. Shoot Parameters

**Figure 1:**
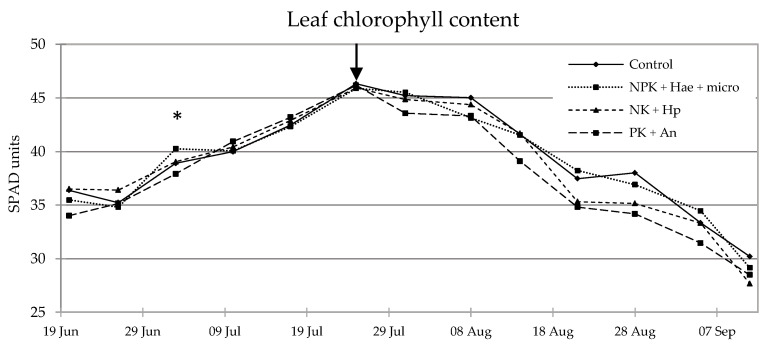
Dynamics of leaf (last fully expanded) chlorophyll content expressed as SPAD (Soil Plant Analysis Development) units (n = 4) in maize plants of a male-sterile inbred line after foliar spraying with three commercial fertilizers/biostimulants, i.e., NPK + Hae + micro, NK + Hp, and PK + An, at V5 stage (16 June), in comparison with the Control. Asterisks indicate significant differences between treatments for each date of measurement (Student–Newman–Keuls test, *p* ≤ 0.05). The arrow indicates the time of silking (25–28 July).

**Figure 2:**
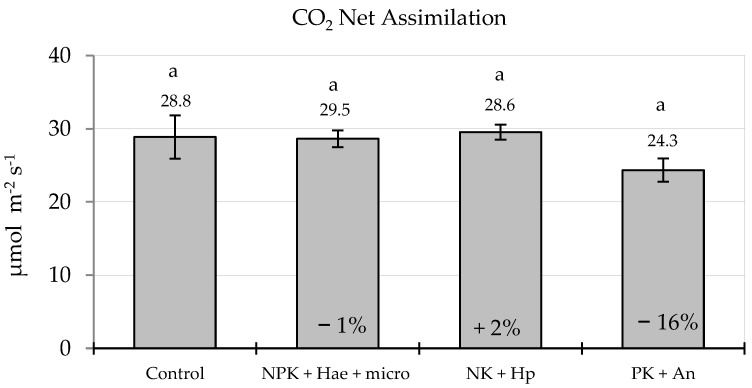
Leaf (last fully expanded) CO_2_ Net Assimilation measured with LICOR 6800 at 31 days after treatment (DAF) (means ± S.E.; n = 4) in maize plants of a male-sterile inbred line after foliar spraying with three commercial fertilizers, i.e., NPK + Hae + micro, NK + Hp, and PK + An, i.e., at V10 stage (17 July), in comparison with the Control. Numbers above histograms indicate mean values; numbers inside histograms indicate the percentage variation of treatments vs. the Control, and different letters indicate significant differences among treatments (Student–Newman–Keuls test, *p* ≤ 0.05).

The dynamics of SPAD indicated that the application of the three fertilizers/biostimulants did not lead to any significant improvement in leaf chlorophyll content during the vegetative phase in the inbred line. The only exception was given by NPK + Hae + micro, which allowed for higher values two weeks after treatment (+34% vs. Control (C); *p* ≤ 0.05) (Figure 1). From mid-August onwards, a decrease in SPAD across all treatments demonstrated the natural senescence dynamics of the flag leaf, with no significant effects on the stay green by any treatment. This was reflected in the CO_2_ net assimilation in the youngest leaf on July 17 at 31 days after treatment (DAT) (V10 stage), which was unaffected by the foliar treatment, although a slight but statistically insignificant increase in assimilation was observed with NK + Hp (+2% vs. C) and a slight and insignificant decrease with PK + An (−16% vs. C; n.s.) (Figure 2).

Even though above-ground biomass increased by 23% with the NK + Hp treatment (26.5 vs. 21.6 g dry weight (DW) plant^−1^ of Control; 29 DAT), there were no statistically significant variations among treatments. Plant height was not affected by the treatments either (trial average, 1.72 m).

### 2.2. Root Parameters

**Figure 3:**
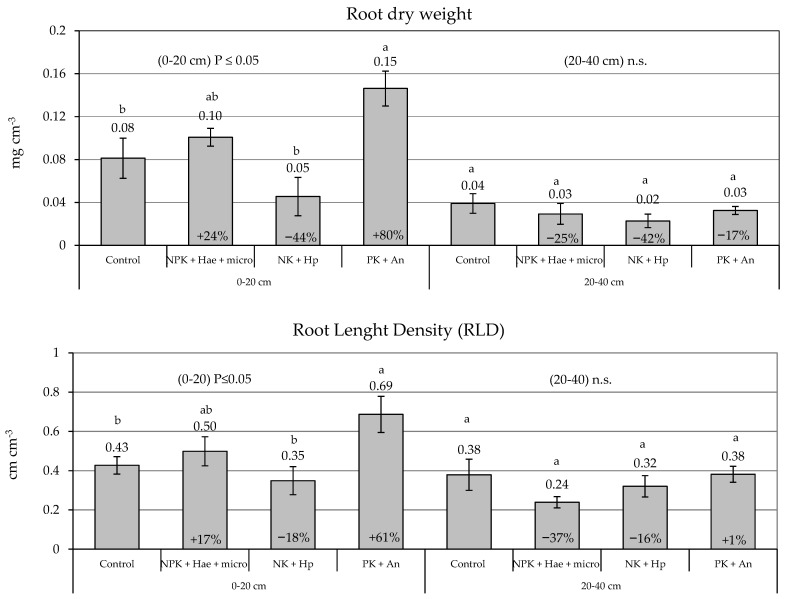
Volumetric root dry weight (mg cm^−3^) (**top**) and root length density (RLD, cm cm^−3^) (**bottom**) at 29 days after treatment (DAF), in two soil layers (0–20 cm and 20–40 cm) (means ± S.E.; n = 4) in maize plants of a male-sterile inbred line after foliar spraying with three commercial fertilizers, i.e., NPK + Hae + micro, NK + Hp and PK + An, at V10 stage (15 July), in comparison with the Control. For each parameter, numbers above histogram indicate means, numbers inside histograms indicate the percentage variation in treatments vs. Controls, and different letters indicate significant differences between treatments (Student–Newman–Keuls test, *p* ≤ 0.05).

All the measured root parameters, except diameter, significantly increased with the PK + An treatment along the whole 0–40 cm soil profile, with improvements of 23% in root area density (RAD) and 48% in root dry biomass (Table 1). This root biostimulant effect was particularly evident in the top 20 cm soil layer, where root dry biomass and root length density (RLD) improved by 80 and 61% with the PK + An treatment, respectively (Figure 3). A beneficial effect of NPK + Hae + micro in the top 20 cm soil layer was also observed and tended towards significant (*p* ≤ 0.1); in these plants, root biomass and length density increased by 23 and 17% vs. Control, respectively. On the contrary, the NK + Hp treatment reduced root expansion in both soil layers (Figure 3), although not significantly.

The two main fixed effects, ‘treatment’ (T; *p* ≤ 0.05) and particularly ‘soil layer’ (SL; (*p* ≤ 0.01), were statistically significant in explaining RLD and RAD as well as root dry biomass. Their interaction (T × SL) was significant (*p* ≤ 0.05) for root dry weight and surface area due to the negative impact of NK + Hp (Table 1).

Measurement of root electrical capacitance (at 120 Hz frequency) taken in parallel with soil corings (29 DAT) corroborated the positive effect of PK + An treatment on root growth, although with a lower and not significant variation (+13% vs. C) as compared with the morphological root measurements. NPK + Hae + micro and NK + Hp treatments allowed for a slight, albeit not significant, improvement in root electrical capacitance, i.e., up to +8% vs. C, in contrast to the results from the soil corings of NK + Hp treated plants (Figure S1).

### 2.3. Yield, Caliber, and Germinability of Kernels

**Figure 4:**
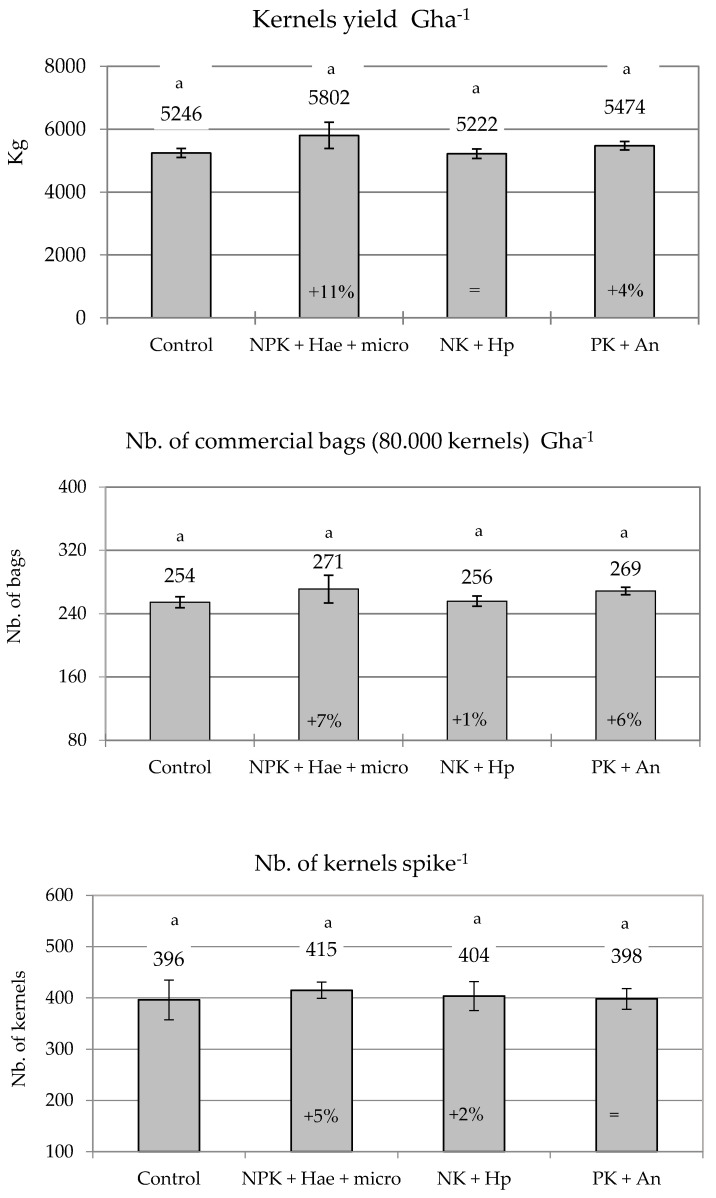
Yield of kernels (Kg DW; **top**) and number of commercial seed bags (of 80,000 kernels each; **middle**) per Gha and number of kernels per spike (**bottom**) (mean ± S.E.; n = 4) following the harvesting of maize ears in a male-sterile inbred line after foliar spraying with three commercial fertilizers, i.e., NPK + Hae + micro, NK + Hp, and PK + An, in comparison with the Control (C). Numbers above histograms indicate the mean; numbers inside histograms indicate the percentage variation in treatments vs. C, and different letters indicate significant differences among treatments (Student–Newman–Keuls test, *p* ≤ 0.05).

At harvest, kernel yield (weight) per Gha showed slight but not significant improvements with the NPK + Hae + micro and PK + An treatments, i.e., +11 and +4% vs. C (5246 Kg of dry kernels Gha^−1^; *p* ≥ 0.05), whereas no differences were associated with the NK + Hp treatment (Figure 4). The effects of the tested fertilizers/biostimulants were seldom further improved when the number of commercial seed bags (one bag equaling 80,000 kernels of commercial size large (L) and medium (M)) per Gha was considered, with a slight but not significant increase observed with NPK + Hae + micro and PK + An treatments, allowing 17 (+7%) and 15 (+6%) additional bags per Gha vs. C, respectively (*p* ≥ 0.05) (Figure 4).

The thousand kernel weight (trial average of 281 g) and the number of kernels per spike (trial average of 403) were not affected by foliar treatments, although the NPK + Hae + micro and NK + Hp treatments increased the number of kernels per spike (+5% and +2%, respectively, vs. C; *p* ≥ 0.05) (Figure 4).

**Figure 5:**
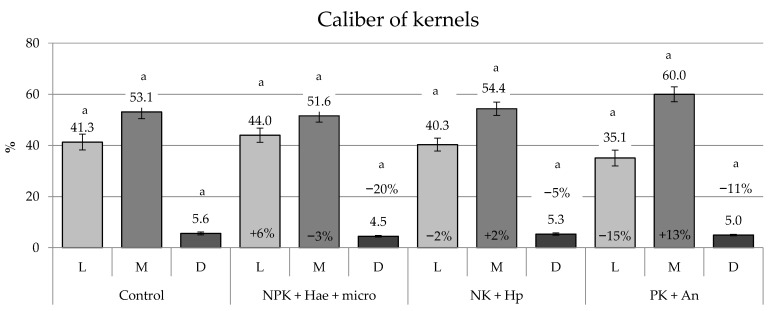
Percentage of kernels with large (L, i.e., 8.0–9.4 mm) and medium (M, i.e., 6.5–8.0 mm) caliber, and percentage of discarded kernels (D, i.e., <6.5 mm or >9.4 mm) (mean ± S.E.; n = 4), in maize plants of a male-sterile inbred line after foliar spraying with three commercial fertilizers, i.e., NPK + Hae + micro, NK + Hp, and PK + An, in comparison with the Control (C). Numbers above histograms indicate means; numbers inside histograms indicate the percentage variation in treatments vs. C; and different letters indicate significant differences between treatments (Student–Newman–Keuls test, *p* ≤ 0.05).

Regarding the commercial caliber of kernels, the percentage of L and M kernels did not vary significantly among treatments (Figure 5). However, an increase of kernels with caliber M (+13% vs. C) at the expense of caliber L (−15% vs. %) was observed in the ears of plants fertilized with PK + An, while a lower and opposite trend was observed in plants treated with NPK + Hae + micro (*p* ≥ 0.05).

Interestingly, the percentage of discarded, i.e., non-marketable kernels conforming neither to the L nor M classes, was reduced with all investigated fertilizers/biostimulants, but only the NPK + Hae + micro treatment significantly reduced the percentage of discarded kernels (−20% vs. C; *p* ≤ 0.05) (Figure 5).

As expected, the germination rate of kernels was generally higher in warm than in cold temperatures (99% vs. 95% in C). It was not a trait that was significantly affected by treatment, and rates were always >90% for both temperatures regardless of the treatment or lack thereof (Table 2).

## 3. Discussion

The present study innovatively investigated the effects of early foliar application of three mixed mineral fertilizers and organic biostimulants on selected physiological and productive parameters in a male-sterile inbred line of maize. The aim was to verify their potential biostimulant properties and benefits on plant morpho-physiology and performance in order to better counteract the low vigor typical for inbred maize lines, while simultaneously ensuring high kernel quality within commercial standards.

The biostimulant properties of PK + An and NPK + Hae + micro were clearly evident at root level. These two fertilizers contained organic carbon from *Ascophillum nodosum* algae and animal epithelium, respectively. Contrary to expectation, no differences or even slight reductions of root expansion in comparison to the Control were observed with the use of NK + Hp, which contained humified peat. Humified peat contains humic and fulvic acids [21,22], both of which have generally been shown to stimulate root growth and plant metabolism [23]. As a source of nutrients, the two most effective products for root growth enhancement both contained P and K, two macro-nutrients recognized for their stimulant effects on root elongation and branching (particularly P) in many crops. This effect was observed in the top 20 cm soil layer in the present study and was associated with a reduction in root diameter. The development of thinner roots allows plants to increase their specific root length for better soil exploration and more efficient nutrient absorption [24].

The product containing *Ascophyllum nodosum* alga extract (PK + An) demonstrated the greatest root biostimulating effect, which was greater than that observed with the use of just P and K, also present in the two other commercial fertilizers/biostimulants. The beneficial effects of algae extracts on root growth of maize have previously been observed by Jeannin et al. [25] and Shukla et al. [26]. Jeannin et al. [25] used *A. nodosum* seaweed cream at different concentrations as a foliar applicant in potted maize plants. The said authors demonstrated an enhancement of root dry biomass with *A. nodosum* foliar application, and this was associated with a stronger sink capacity of the root apparatus. Increased root elongation, particularly of the finest roots, and greater root surface area together improved nutrient uptake, increased esterase activity, and elevated sugar content, as recently documented by Ertani et al. [27], who supplied different algae extracts to the soil of maize seedlings. Although the biostimulating properties of organic mineral fertilizers are often highly interesting at a subterranean level, such fertilizers struggle to involve the shoot [28], which was similarly mirrored in the results of the current study. Indeed, the lack of relevant benefits conferred by the tested fertilizers/biostimulants on some key physiological parameters related to photosynthesis, such as leaf chlorophyll content and net CO_2_ assimilation, is documented here. Accordingly, no evident changes in shoot height or biomass were recorded in treated plants.

However, positive root responses with PK + An and NPK + Hae + micro treatments were associated with a moderate increase in the number of commercial seed bags per Gha. This was due to a slight increment in the number of kernels per spike under NPK + Hae + micro treatment, possibly related to its higher K content and micronutrients, such as zinc, which is recognized to be involved in phytohormone activity, seed production, carbohydrate metabolism, and protein synthesis [29]. The amount of nutrients supplied by foliar spraying was limited compared to the plant requirements, and certain factors such as climatic conditions, cultivation method, and mode and timing of application may affect the efficacy of foliar treatment in the trials of the current study, particularly aboveground. The climatic conditions during the growing season in 2020 were unfavorable for maize growth, and these stress-related conditions may indeed have reduced the efficacy of the treatments, as hypothesized by Stefanović et al. [30]. In this regard, low temperatures and abundant rainfall were documented during the whole month of June, which caused a delay in phenology and reduced plant height and development as compared with the standard values for the female inbred line used in the present study (1.72 m vs. a common value of 2.1–2.2 m). Additional drought stress conditions in July and August were further detrimental to crop growth, despite adequate irrigation. Similarly, in a recent study by Brankov et al. [31], the lack of relevant seed yield improvements in an inbred line of maize following the application of two fertilizers containing macro- and microelements and amino acids was associated with adverse climatic conditions. The low efficacy of foliar application of fertilizers was ascribed to low precipitation and a highly heterogeneous rainfall pattern by Pecha et al. [32] and Pereira et al. [28]. According to these authors, the moderate effects on seed production of the fertilizers/biostimulants tested in the current study could be due to multiple abiotic stress conditions. The low vigor of inbred maize line plants might be further impaired by herbicide application as these lines are particularly sensitive to certain a.i. The female inbred line used in the present study is known to be slightly sensitive to the a.i. s-metolachlor, which was present in the pre-emergence treatment applied in this trial.

The choice of application timing is one of the main factors influencing foliar treatment efficacy. Fertilizer application at the V5 stage was not associated with a relevant increase in yield, even though Brankov et al. (2020) had previously identified this stage as the optimal one for maximizing biomass, leaf area index, and kernel productivity in comparison to later application timing at 11–12 leaves [31]. Brankov et al. [31] tested biostimulant commercial products containing amino acids. Similarly, a recent study on the effects of foliar-applied biostimulants extracted from the seaweed *Ascophyllum nodosum* in maize at different time points between the V8 (eight-leaf) and R1 (silking) stages reported a lack of evident improvement in plant performance [28]. Maize commonly starts to differentiate the florets at the V6 (six-leaf) stage, while the number of ranks per ear is determined by the V12 (twelve-leaf) stage [33,34]. Optimal conditions are crucial in this time interval for achieving high kernel yield and require well-nourished plants, free from biotic and abiotic stresses that can otherwise compromise the future number of kernels per ear. As such, the application of organic-mineral fertilizers/biostimulants at V5–V6, like in the present study, would be expected to improve the plant’s nutritional status and plant wellbeing, with resulting improvements in kernel productivity. Here, an overall moderate decrease in the percentage of discarded kernels due to a non-marketable caliber and the lack of significant changes in germination rates represent a positive achievement independent of fertilizer/biostimulant choice.

Nitrogen nutrition has a specific role in floret differentiation. An appropriate N rate can both increase N metabolism-related enzymatic activity and increase the glutamine and asparagine contents of leaves [35]. This, in turn, increases N transfer to the spikes, promoting spike differentiation and decreasing abortive florets [35]. Although moderate, the improved ear fertility by PK + An application was therefore an unexpected observation in the current study, as this fertilizer does not contain mineral N. It can more likely be explained by the positive effects of the algae extracts, which compensated for the lack of mineral N. The combination of the three macro-nutrients NPK, micronutrients, and hydrolyzed epithelium can maximize the profitability of seed production and commercialization due to the improved percentage of large kernels, which are better handled by the pneumatic seeder.

Animal epithelium and animal waste in general represent a rich source of biodegradable C, N, H, O, and S. As opposed to utilizing the direct proteinaceous by-product associated with the livestock and poultry industries, enzymatic, chemical, and chemical-enzymatic hydrolysis of these, as was done for the epithelium content of the NPK + Hae + micro treatment in the current study, provides peptides of varying lengths as well as free amino acids [36]. Indeed, besides providing essential nutrients, protein hydrolysates have been shown to possess improved functional (i.e., solubility; [37]) and bioactive (i.e., mimicry of phyto-hormones; [38]) properties, which may promote bioavailability and improve crop performance. Ertani et al. [39] demonstrated that various maize genotypes experienced increased root and shoot growth, as well as elevated concentrations of micronutrients in the roots upon feeding meat hydrolysates from tanning by-products that contain animal epithelium. This can be hypothesized to translate into improved kernel yield, although this was not a trait studied in their trial. Proteins of animal origin possess a diversity of hydrophobic and polar amino acids, unlike plant-derived proteins, which create a background for numerous possible protease cleavage sites [38]. This produces a unique mix of peptides with different chemical properties and, therefore, functionalities from plant hydrolysates [40]. The inclusion of hydrolyzed animal epithelium in biostimulant formulations may thus greatly benefit the maize seed production industry.

## 4. Materials and Methods

### 4.1. Field Trial Set-Up

The trial was carried out in 2020 on a male-sterile maize inbred line used as a seedbearing line for hybrid seed production (FAO class 700) at the “Ca’ Corniani” farm located at Caorle (Venice, NE Italy, 45°61′ N, 12°82′ E, 2 m a.s.l.). The site was characterized by silty-clay-loamy soil, with the main characteristics described in Table 3, as revealed by the analyses carried out at the laboratories of Labs & Technological Services AGQ, S.L. (Sevilla, ES, Spain).

Beyond low vigor, the studied seedbearing inbred line also suffers from leaf firing, which is responsible for early necrosis of leaf margins and consequent loss of photosynthetic capacity [41].

Following local recommendations, 85 kg ha^−1^ P_2_O_5_ and 55 kg ha^−1^ K_2_O were incorporated into the soil prior to sowing via harrowing. Sowing took place at the beginning of May at intervals of 22 cm along rows 75 cm apart, resulting in roughly 60,000 plants ha^−2^. The plots were dress-fertilized in mid-June with 230 kg N ha^−1^ in the form of urea, which was incorporated into the soil by means of interrow tillage. The a.i. Chlorantraniliprole and Lambda-Cyhalothrin were sprayed in mid-July to protect against the European corn seed borer (*Ostrinia nubilalis*) and later (at silking time, i.e., 25 July) with Indoxacarb to deter the Western corn rootworm (*Diabrotica virgifera*). Weed control was ensured by spraying the a.i. Mesotrione, S-Metolachlor, and Terbutilazina as a pre-emergence treatment just after sowing, and the a.i. Mesotrione and Nicosulfuron as a post-emergence treatment at the beginning of June.

Three commercial mixed mineral fertilizers and organic biostimulants, here named (*i*) NPK + Hae + micro, (*ii*) NK + Hp, and (*iii*) PK + An, were applied to the male-sterile inbred line at the V5 stage by means of foliar spraying (16 June; Table 4) and were grown beside a control treatment C (Table 4). The fertilizers were applied during mid-day at the recommended dosage, i.e., 1 kg ha^−1^, 1.5 L ha^−1^, and 4 L ha^−1^ for NPK + Hae + micro, NK + Hp, and PK + An, respectively. The foliar treatment was applied using a manual sprayer bar with a water volume of 300 L ha^−1^, while C was never sprayed, not even with water. The trial was arranged following a Latin square design with 4 replicates (n = 4), resulting in a total of 16 plots. Each plot/replica measured 4.5 m across (6 rows of seedbearing maize plants) and 10 m along, for a total area of 45 m^−2^. Between each column/replicate plot, there were two rows of male maize line plants spaced 0.5 m apart.

During June 2020, when the fertilizers were applied, the climatic data (monthly rainfall and mean temperature) revealed lower temperatures (−1.3 °C) and abundant rainfall (229 mm vs. 78 mm), compared with the historical 10-year means (Figure S2). Conversely, July witnessed lower rainfall (34 mm vs. 65 mm) and temperature (−0.8 °C) in relation to historical means. In August, the climatic data revealed slightly higher precipitation (99 mm vs. 72 mm) and an average temperature comparable with the 10-year historical mean (~23.4 °C) (Figure S2). In order to contrast the negative effects of water stress, plants were irrigated four times by means of a pivot system between early-July and mid-August, for a total of 100 mm.

### 4.2. Plant Physiological Parameters

The leaf chlorophyll content was indirectly quantified as SPAD units, measured weekly on the last fully expanded leaf of five tagged plants randomly chosen in each plot/replicate, starting from one week after foliar application (V5 stage) until leaf senescence. This was done using a Soil Plant Analysis Development (SPAD)-502 chlorophyll meter (Konica-Minolta, Hong Kong, China) [42]. Three measurements were taken on each leaf: one one-fourth the distance to the leaf apex; one one-fourth the distance to the leaf collar; and one midway up the length of the leaf.

Net CO_2_ assimilation (A) was measured 31 DAT (17 July), on the last fully expanded leaf by means of an infrared gas analyzer LI-6800 (Li-COR Inc., Lincoln, NE, USA). The measurement was undertaken on one plant per replicate per treatment. In order to fully evaluate the potential of photosystem II (PSII), the instrument was set with a pulsed actinic far-red light at 8000 µmol m^−2^ s^−1^ which ensured full excitation of photosystem I (PSI), thus allowing for complete discharge of electrons from PSII, thereby avoiding the initial dark adaptation of the leaf. The temperature of the leaf chamber was set at 27 °C, the relative humidity at 51%, and the air flow at 500 µmol s^−1^. The reference CO_2_ level was set at 400 μmol mol^−1^ and the photosynthetic photon flux density (PPFD) at 1500 µmol m^−2^ s^−1^ (97% light red; 3% blue). Net CO_2_ assimilation (A) was determined following Murchie et al. [43].

### 4.3. Plant Height and Biomass

Plant height was measured weekly on the same five plants per plot (replicate) chosen for SPAD measurements. Plant height was taken from the soil surface until the latest visible leaf collar. Shoot dry biomass was determined at 29 DAT on 15 July, i.e., at the V10 stage, on one plant (cut at crown level) per plot (n = 4), after oven-drying at 105 °C for 48 h.

### 4.4. Root Growth Analysis

The dynamics of root growth were monitored from the time of foliar treatment until the end of July by non-destructive measurement of the root electrical capacitance. Data were acquired weekly by a handheld digital electrical capacitance meter (ELC-131D, Escort Instruments Corporation, Taipei, Taiwan), following the methods described in Romdhane et al. [44]. Electrical capacitance measurements are reported to be highly correlated with root surface area, weight, and activity (i.e., root surface in contact with soil water), as root cells exert considerable resistance to a current passing through the root system [45]. Measurements were carried out at both 1 and 0.12 kHz frequencies.

To investigate the main root parameters, one soil core (50 mm diameter, 40 cm deep) was collected at 29 DAT on July 15 (V10 stage) per plot. The soil cores were collected using a core sampler at a distance of 5 cm from the crown of a maize plant in the inter-row. Each soil core was split into 20 cm deep sub-samples (i.e., 0–20 and 20–40 cm) in order to effectively describe the rooting profile. Roots were separated from the soil particles by flotation with a hydraulic centrifugation device and collected in a 500 μm mesh sieve to be stored at 4 °C in an ethanol solution (12% *v*/*v*) before analysis [46]. Images of roots were subsequently acquired by digital scanning, and measurements of root morphological parameters, i.e., volumetric root length density (RLD), root area density (RAD), and root diameter (D), were performed following the procedure described in Vamerali et al. [47].

To quantify biomass, roots from subsamples were weighed after oven-drying at 105 °C for 36 h.

### 4.5. Hybrid Seed Yield and Yield Components

Hybrid seed yield was measured on September 18 by collecting the ears over an area of 2.5 m^−2^ in each plot. The dry weight of kernels per ear, per plant, and per gross hectare (1 Gha = one hectare of maize plants cultivated for seed production, where 84% of the surface hosts female seedbearing plants, and 16% male pollinating plants), and the number of kernels per unit seed weight (1 kg) were determined. The number of seed bags per Gha, each containing 80,000 kernels, was also determined for every sample considering the number of kernels per ear, the plant density (6 plants m^−2^), the surface area occupied by female plants (8400 m^−2^ ha^−1^), and the percentage of discarded kernels (i.e., non-marketable kernels that did not fit the caliber standards defined below).

For each sample, the kernels were subdivided into two classes according to their caliber: large kernels (L), i.e., with a caliber between 8.0 and 9.4 mm, and medium-large kernels (M), i.e., with a caliber between 6.5 and 8.0 mm. The procedure was carried out by means of sieves with different mesh sizes according to the above thresholds. The kernels fitting neither the L nor M classes, that is, with a caliber <6.5 mm or >9.4 mm, were discarded as non-marketable kernels, and the percentage of discard kernels was calculated for each sample.

### 4.6. Seed Germinability

For each treatment, the germination rate was determined for both caliber classes on 4 replicates consisting of 100 kernels each. Kernels were sown in a substrate of sterilized siliceous sand with a particle diameter of 0.05 to 0.08 mm, and a pH between 6.5 and 7.5 and kept in growth chambers. Germination was tested at two different temperatures, i.e., at a high temperature (25 °C) and a low temperature (12 °C). The first count of germinated kernels was carried out at 4 days after sowing (DAS), and the second and final counts were carried out at 7 DAS following the ISTA (Internation Seed Testing Association, Wallisellen—CH) protocol.

### 4.7. Statistical Analysis

The data from all the assessed parameters were subjected to ANOVA with covariance analysis using Costat software (Cohort software, Manugistics, Rockville, MD-USA; ver. 6.204). Separation of means was set at *p* ≤ 0.05 with the Student–Newman–Keuls test.

## 5. Conclusions

Foliar spraying represents a method with great potential for improving seed yield and quality in hybrid seed production in maize. The present study demonstrated that male-sterile inbred lines of maize could benefit from early foliar application of mixed mineral fertilizers and organic biostimulants, which partly reached the soil at the five-leaf stage of the plants. Products containing P, as well as K and/or algae extracts, exert a highly evident biostimulating effect in the roots of the shallow soil layers. This biostimulant effect can be translated into greater kernel numbers and, therefore, improved kernel productivity and a lower fraction of discharged kernels. However, optimal improvements in terms of the number of commercial seeds can be obtained when nutrients are combined with hydrolyzed animal epithelium, as in the fertilizer NPK + Hae + micro. Among nutrients, mineral N should be further included in fertilizer/biostimulant formulations, together with P and K and possibly some micronutrients such as Zn, to reach optimal effects. Nonetheless, root amelioration itself might be a premise for better counteracting the low vigor of inbred lines. However, much remains to be elucidated to enhance the application protocol for foliar application of these products. Confirmation of results in other growing seasons, as well as investigations into different formulations and doses, the number of applications, and the application timing within a longer experimental timespan containing later growth stages, is crucial. Such investigations are also necessary to ascertain the effects of foliar treatments under mechanical emasculation of male-fertile seedbearing inbred maize lines, with expected greater effects.

## Figures and Tables

**Table 1:** Root dry biomass, root length density (RLD), root area density (RAD), and diameter (D) in the 0–40 cm soil layer (mean ± S.E. and analysis of variance; n = 4) in maize plants of a male-sterile inbred line at 29 days after foliar spraying of three commercial fertilizers, i.e., NPK + Hae + micro, NK + Hp, and PK + An, at V10 stage (15 July), in comparison with the Control (C). Within the same root parameter, the percentage of variation vs. C is reported, while different (superscript) letters within the same column indicate significant differences among treatments according to the Student–Newman–Keuls test (*p* ≤ 0.05).

	Root Dry Weight (g cm^−3^)		RLD (cm cm^−3^)		RAD (cm^2^ cm^−3^)		D (µm)	
Factors	Mean	% var. vs. C	Mean	% var. vs. C	Mean	% var. vs. C	Mean	% var. vs. C
Control	0.060 ^ab^		0.403 ^ab^		0.064 ^ab^		996 ^a^	
NPK + Hae + micro	0.061 ^ab^	+2%	0.368 ^ab^	−9%	0.058 ^ab^	−9%	948 ^a^	−5%
NK + Hp	0.037 ^b^	−38%	0.334 ^b^	−17%	0.045 ^b^	−30%	886 ^a^	−11%
PK + An	0.089 ^a^	+48%	0.534 ^a^	+33%	0.079 ^a^	+23%	910 ^a^	−9%
Treatments (T)	-	**	-	*	-	*	-	n.s.
Soil Layer (SL)	-	***	-	**	-	***	-	***
T × SL	-	**	-	n.s.	-	*	-	n.s.

ns: not significant; *: significant at *p* ≤ 0.05; **: significant at *p* ≤ 0.01; ***: significant at *p* ≤ 0.001.

**Table 2:** Percentage of germinated kernels under warm and cold conditions (mean ± S.E.; n = 4). Kernels were harvested from maize ears in a male-sterile inbred line after foliar spraying with three commercial fertilizers, i.e., NPK + Hae + micro, NK + Hp, and PK + An, in comparison with the control. For the same temperature, the percentage of variation in treatments vs. Control is reported. Different letters indicate significant differences among treatments within the same temperature (Student–Newman–Keuls test, *p* ≤ 0.05).

	Germination Rate (%)			
Treatments	Warm (25 °C)		Cold (12 °C)	
Control	98.9 ± 0.6 ^a^		94.6 ± 2.9 ^a^	
NPK + Hae + micro	99.5 ± 0.2 ^a^	+1%	91.3 ± 2.5 ^a^	−3%
NK + Hp	99.2 ± 0.5 ^a^	=	94.0 ± 1.6 ^a^	−1%
PK + An	96.9 ± 2.2 ^a^	−2%	92.1 ± 2.7 ^a^	−3%

**Table 3:** Soil characteristics at the study site.

Soil Characteristics	Value
Clay	27%
Silt	57%
Sand	16%
pH	8.49
Organic matter	1.75%
Total N	953 mg kg^−1^
Assimilable P_2_O_5_	19.7 mg kg^−1^
Exchangeable K_2_O	152 mg kg^−1^

**Table 4:** Code, type of formulation, and composition of three organic-mineral fertilizers/biostimulants (“m.v.” missing value).

Fertilizer/Biostimulant Code (Type of Formulation)	Composition	Value (%)
NPK + Hae + micro (powder)	Organic C (from hydrolyzed animal epithelium)	7.5
	Total N:	10.0
	N-NH_3_	6.0
	N-NO_3_	2.5
	Organic N	1.5
	Total P_2_O_5_:	5.0
	P_2_O_5_ soluble in neutral ammonium citrate	5.0
	P_2_O_5_ soluble in water	5.0
	Water-soluble K_2_O	17.0
	Water-soluble SO_3_	23.0
	Water-soluble MgO	5.0
	Fe-EDTA	0.2
	Mn-EDTA	0.1
	Mo	0.001
	Zn-EDTA	0.1
NK + Hp (liquid suspension)	Organic C (from humified peat)	8.7
	Total N	13.0
	Organic N	0.5
	Ureic N	12.5
	Water-soluble K_2_O	5.0
PK + An (liquid)	Organic C (from *Ascophyllum nodosum*)	m.v.
	Total P_2_O_5_	13.0
	Water-soluble K_2_O	5.0

## Data Availability

The data presented in this study are available on request from the corresponding author.

## Supplementary Materials

The following supporting information can be downloaded at: https://www.mdpi.com/article/10.3390/plants12152837/s1, Figure S1: Root electrical capacitance (at 0.12 kHz frequency) at 29 days after treatment (DAT) in the top 0–55 cm soil layer (mean ± S.E.; n = 4) in maize plants (two plants per plot/replicate among the five plants previously chosen for SPAD measurements) of a male sterile inbred line after foliar spraying with three commercial fertilizers, i.e. NPK + Hae + micro, NK + Hp and PK + An, at V10 stage (15 July), in comparison with the Control (C). Numbers above histograms indicate means, number inside histograms indicate the percentage variation in treatments vs. C, and different letters indicate significant differences between treatments (Student–Newman–Keuls test, *p* ≤ 0.05); Figure S2: Monthly mean temperature and precipitation across the growth cycle of maize at the closest weather station to the trial site at Eraclea (Venice, Italy).
