# Supplementary material for: Effects of Foliar-Applied Mixed Mineral Fertilizers and Organic Biostimulants on the Growth and Hybrid Seed Production of a Male-Sterile Inbred Maize Line
Source: Plants (Basel). 2023 Jul 31;12(15):2837. doi: 10.3390/plants12152837 (PMC10421008; doi:10.3390/plants12152837)
Supplement: Supplementary file 1 [file plants-12-02837-s001.zip › plants-2448209-supplementary.pdf]

SUPPLEMENTARY INFORMATION

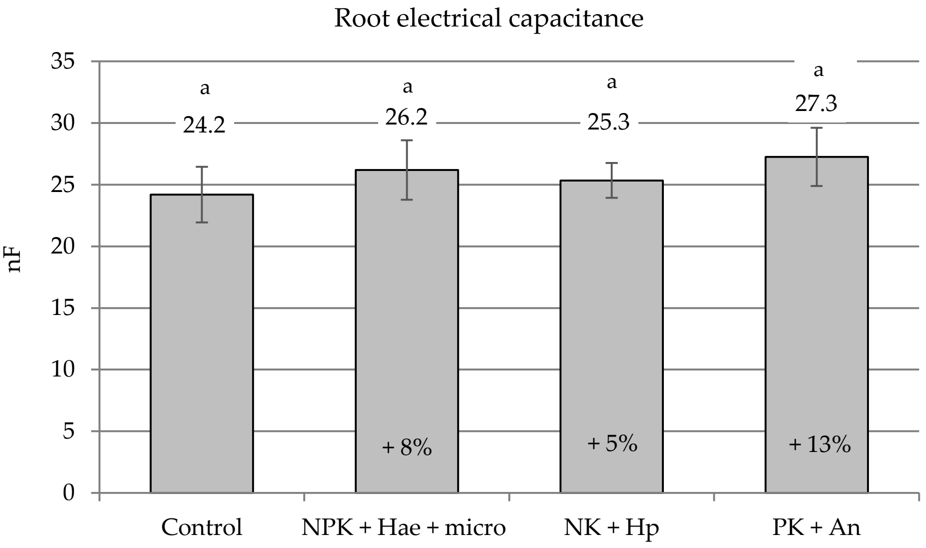

Figure S1. Root electrical capacitance (at 0.12 kHz frequency) at 29 days after treatment (DAT) in the top 0-55 cm soil layer (mean ± S.E.; n=4) in maize plants (two plants per plot/replicate among the five plants previously chosen for SPAD measurements) of a male sterile inbred line after foliar spraying with three commercial fertilizers, i.e. NPK + Hae + micro, NK + Hp and PK + An, at V10 stage (15 July), in comparison with the Control (C). Numbers above histograms indicate means, number inside histograms indicate the percentage variation in treatments vs. C, and different letters indicate significant differences between treatments (Student–Newman–Keuls test, P≤0.05).

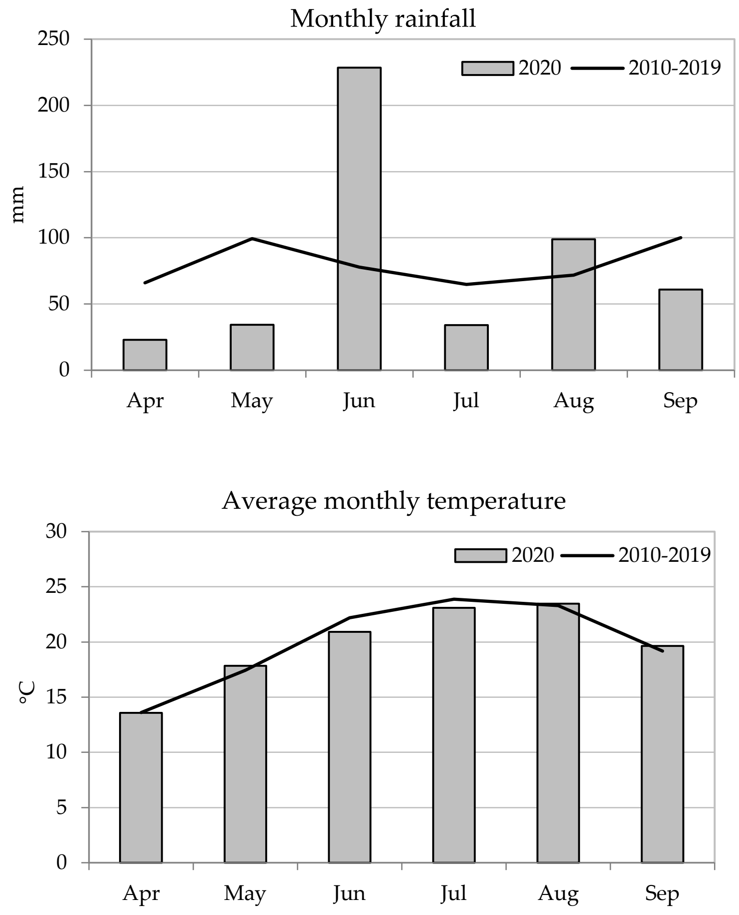

Figure S2. Monthly mean temperature and precipitation across the growth cycle of maize at the closest weather station to the trial site at Eraclea (Venice, Italy).
